# Identification of quantitative trait loci and development of diagnostic markers for growth habit traits in peanut (*Arachis hypogaea* L.)

**DOI:** 10.1007/s00122-023-04327-9

**Published:** 2023-04-07

**Authors:** Yuanjin Fang, Xinyou Zhang, Hua Liu, Jihua Wu, Feiyan Qi, Ziqi Sun, Zheng Zheng, Wenzhao Dong, Bingyan Huang

**Affiliations:** 1 College of Agriculture, Nanjing Agricultural University, Nanjing, 210095 China; 2 Henan Academy of Agricultural Sciences, Henan Institute of Crop Molecular Breeding, Shennong Laboratory, Key Laboratory of Oil Crops in Huang-Huai-Hai Planis, Ministry of Agriculture, Henan Provincial Key Laboratory for Oil Crops Improvement, Zhengzhou, 450002 China; 3 Shangqiu Academy of Agriculture and Forestry, Shangqiu, 476002 China

**Keywords:** Peanut, QTL, Growth habit, Molecular markers, Pod number

## Abstract

**Key message:**

QTLs for growth habit are identified on Arahy.15 and Arahy.06 in peanut, and diagnostic markers are developed and validated for further use in marker-assisted breeding.

**Abstract:**

Peanut is a unique legume crop because its pods develop and mature underground. The pegs derive from flowers following pollination, then reach the ground and develop into pods in the soil. Pod number per plant is influenced by peanut growth habit (GH) that has been categorized into four types, including erect, bunch, spreading and prostrate. Restricting pod development at the plant base, as would be the case for peanut plants with upright lateral branches, would decrease pod yield. On the other hand, GH characterized by spreading lateral branches on the ground would facilitate pod formation on the nodes, thereby increasing yield potential. We describe herein an investigation into the GH traits of 521 peanut recombinant inbred lines grown in three distinct environments. Quantitative trait loci (QTLs) for GH were identified on linkage group (LG) 15 between 203.1 and 204.2 cM and on LG 16 from 139.1 to 139.3 cM. Analysis of resequencing data in the identified QTL regions revealed that single nucleotide polymorphism (SNP) or insertion and/or deletion (INDEL) at Arahy15.156854742, Arahy15.156931574, Arahy15.156976352 and Arahy06.111973258 may affect the functions of their respective candidate genes, *Arahy.QV02Z8*, *Arahy.509QUQ*, *Arahy.ATH5WE* and *Arahy.SC7TJM*. These SNPs and INDELs in relation to peanut GH were further developed for KASP genotyping and tested on a panel of 77 peanut accessions with distinct GH features. This study validates four diagnostic markers that may be used to distinguish erect/bunch peanuts from spreading/prostrate peanuts, thereby facilitating marker-assisted selection for GH traits in peanut breeding.

**Supplementary Information:**

The online version contains supplementary material available at 10.1007/s00122-023-04327-9.

## Introduction

Cultivated peanut (*Arachis hypogaea* L.) is an allotetraploid legume resulting from the spontaneous hybridization of two diploid species in South America, *Arachis duranensis* and *Arachis ipaensis*, followed by natural chromosome doubling (Bertioli et al. 2016). Peanut, commonly known as groundnut, is a unique oilseed crop in which pegs form aboveground after fertilization, extend to the soil through elongation, and pods form and develop underground. Peanut is regarded as an important source of vegetative oil and protein worldwide. China currently ranks first in global peanut production (FAOSTAT2020 https://www.fao.org/faostat/en/#home), which is primarily driven by the rising demand for edible vegetable oil. In the USA, peanuts are processed into peanut butter, roasted peanuts, confectionery products and snack foods. With a growing global population and limited agricultural land, it is imperative to increase peanut productivity to meet the rising demand.

The architecture of a plant, or its growth habit (GH), is an important agronomic trait that affects crop yield and facilitates mechanized farming. The plant architecture of cereal crops, such as plant height, number of tillers and tiller angle, correlates directly with grain quality and yield. During the Green Revolution, “Dwarfing alleles” *Rht* and *sd1* were selected in wheat and rice, respectively, to boost crop productivity (Hedden 2003). Recent research has revealed the genetic mechanisms underlying tiller angle in rice (Hu et al. 2020). QTLs for tiller angle in bread wheat and branch angle in soybean have also been identified (Zhao et al. 2020; Clark et al. 2022).

The GH of peanuts can be classified into four morphological types based on the establishment of the first pair of the lateral branches and the main stem, including erect, bunch, spreading and prostrate (Pittman 1995). Wild *Arachis* species, such as the diploid progenitors of cultivated peanut, *A. duranensis* and *A. ipaensis,* are of the procumbent type, whereas distinct botanical varieties of the cultivated peanut, *A. hypogaea*, exhibit procumbent (prostrate), decumbent (spreading), semi-erect (bunch) and erect GH traits (Krapovickas and Gregory 2007). Botanical groups *var. hypogaea* and var. *hirsuta* belong to subsp. *hypogaea* and display prostrate or spreading traits, whereas accessions in subsp. *fastigiata* display bunch or erect GH traits. The peanut varieties released in China are typically erect or bunch types, with pods clustered at the base of the plant, and mature early, rendering them suitable for the two-crop rotation system. In contrast, peanut cultivars in the USA are primarily prostrate or spreading, demanding wide row spacing and a lengthy growth period (Gorbet and Knauft 1997; Gorbet and Tillman 2009; Simpson et al. 2013; Burow et al. 2014; Branch and Brenneman 2015; Holbrook et al. 2017). For the erect and bunch types of peanuts, the number of pods per plant is often constrained by the number of flowers on lower branches that could easily reach and penetrate the ground. In contrast, the GH trait of the long, prostrate or spreading lateral branches permits more flowers to develop into pods, thereby increasing the number of pods per plant.

The identification of QTL and molecular markers for important traits is crucial for peanut breeding and germplasm utilization. Through genetic studies, molecular markers for oleic acid content and root-knot nematode resistance have been developed and implemented in marker-assisted selections (Janila et al. 2016; Holbrook et al. 2017; Huang et al. 2019). In recent years, QTL mapping studies for rust, late leaf spot, bacterial wilt resistance and seed oil content have been reported, along with the development of molecular markers (Liu et al. 2020; Sun et al. 2022; Pandey et al. 2017; Qi et al. 2022). Recent research has centered on yield-related traits such as seed weight, shelling percentage and maturity. A QTL for seed weight was identified on chromosome B06, enabling the development of its Kompetitive Allele Specific Polymerase Chain Reaction (KASP) marker (Wang et al. 2022). QTLs for shelling percentage were identified on chromosomes A09 and B02 (Luo et al. 2019) and chromosomes A07 and A08 (Li et al. 2021), respectively. QTLs for maturity traits have been discovered on chromosomes A04 and B03 in Virginia-type peanut (Kunta et al. 2021). Likewise, a QTL for peanut GH was identified between 145 and 146 Mbp on chromosome B05 in F_3_ families descended from a cross between two Virginia-type parents (Kayam et al. 2017). More recently, SLAF-seq genotyping of a RIL population consisting of 188 peanut lines revealed a GH QTL interval on chromosome B05 (Li et al. 2019). Nevertheless, as a yield-related trait, GH is governed by multiple genes with various degrees of contribution, rendering its inheritance complex. It is thus desirable to comprehend the genetic control of peanut GH traits on diverse genetic backgrounds. In this study, a RIL population consisting of 521 peanut lines has been developed through a genetic cross between the released variety Yuanza9102 that entails wild *Arachis* germplasm in its pedigree and a peanut germplasm accession wt09-0023 that is rich in oleic acid in its seed oil. Our objectives were to identify QTLs for peanut GH traits in these RILs and to develop molecular markers for selection of different GH traits in peanut breeding.

## Materials and methods

### Plant materials

A population consisting of 521 RILs was derived from a cross between Yuanza9102 and wt09-0023, which was described in detail previously (Qi et al. 2022). Released by Henan Academy of Agricultural Sciences in 2002, Yuanza9102 is a high-yielding Spanish-type peanut that was generated by hybridizing a peanut landrace with a diploid *Arachis* species. The germplasm accession wt09-0023 is a high-oleic breeding line introduced from the USA (AgResearch Consultants Inc., ACI Seeds, Tifton, GA, USA). Yuanza9102 and wt09-0023 are erect and bunch, respectively. A high level of segregation for the GH trait was observed in the derived RIL population. In May 2021, the F_10_ RIL population was planted following a randomized block design in three different growth environments, including Yuanyang (sandy loam soil), Shangqiu (loam soil type) and Nanyang (clay soil type). Each plot consisted of two rows spaced 40 cm apart. Each row measured 150 cm in length with 15 cm between plants. The field management adhered to the standard field practice. Before planting, the field was well irrigated and 450 kg per hectare of compound fertilizer (N:P:K, 14:16:15) was applied. Plants were irrigated to maintain 60–70% soil moisture throughout the entire growing season.

### Investigation of peanut growth habit traits

The GH and index of plant type (IOPT) of 521 RILs were investigated in the field about 60 days after sowing. GH was classified into four morphological types, including erect, bunch, spreading and prostrate based on standards established by Pittman (1995). Before harvesting, ten plants were selected from each line, and the height of the main stem and the length of the first lateral branches were measured. IOPT was calculated by dividing the length of the first lateral branch by the height of the main stem. The distribution of IOPT in each distinct GH type was represented as a violin plot drawn by using Python 3.9. The scatter plots depicting the relationship between IOPT, the height of the main stem and the length of the lateral branch were generated by the R package PerformanceAnalytics 2.0.4.

### QTL analysis

The RIL population was genotyped by Restriction Site-Associated DNA sequencing (RAD-seq). After screening for polymorphic SNPs between the RIL parents and further filtration, 24,852 SNPs remained in the dataset. Redundant markers were then removed, and a genetic linkage map with twenty linkage groups (LG) consisting of 5120 markers was constructed by using Joinmap v5.0 (Van Ooijen 2018) as previously described (Qi et al. 2022). The linkage map was plotted using MapChart 2.32 (Voorrips 2002). QTL mapping was performed by using ICIM-ADD model with a step size of 0.1 and the ICIM-EPI model with a step size of 1.0, implemented in QTL IciMapping v4.2 (Meng et al. 2015).

### Molecular marker development and validation

SNPs and INDELs in the QTL region were examined in the peanut resequencing data. Polymorphic markers between RIL parents were selected, and the effects of these variants were predicted by SnpEff version 5.1 (Cingolani et al. 2012). Three markers for *qGHA15* on chromosome 15 were designed for KASP genotyping that was conducted by China Golden Marker Biotech Co., Ltd. (Beijing, China), and an INDEL marker for *qGHA06* on chromosome 6 was developed by using Primer3Plus (https://www.bioinformatics.nl/cgi-bin/primer3plus/primer3plus.cgi) (Table S1). A 20 μL PCR was prepared, including 4 μL of 5 × buffer, 1.6 μL of dNTP (2.5 mM), 0.4 μL of PrimeSTAR® GXL (Takara Bio Inc.) DNA polymerase (1.25 U/μL), forward primer (10 μM), reverse primer (10 μM) and 5 μL of genomic DNA (50 ng/μL). PCR was performed under following conditions: initial denaturation at 95 °C for 5 min, 35 cycles of denaturation at 95 °C for 10 s, annealing at 57 °C for 15 s, extension at 68 °C for 12 s and a final extension at 68 °C for 7 min. The 200 bp PCR product was sequenced by BGI Tech Solutions Co., Ltd. (Beijing, China). The genomic DNAs of 165 RILs and 77 germplasm accessions were extracted by applying the CTAB method (Murray and Thompson 1980). Markers for *qGHA15* and *qGHA06* were developed to genotype 165 RILs and a validation panel. The validation panel consisted of 77 accessions, including 35 germplasm with a prostrate or spreading GH trait, and 42 peanut accessions with an erect or bunch GH trait. The statistical associations between genotypes and GH trait were represented in boxplot produced from R software version 4.1.2 (R Core Team 2021).

## Results

### Phenotypic variation of peanut growth habit traits in the RIL population

**Fig. 1:**
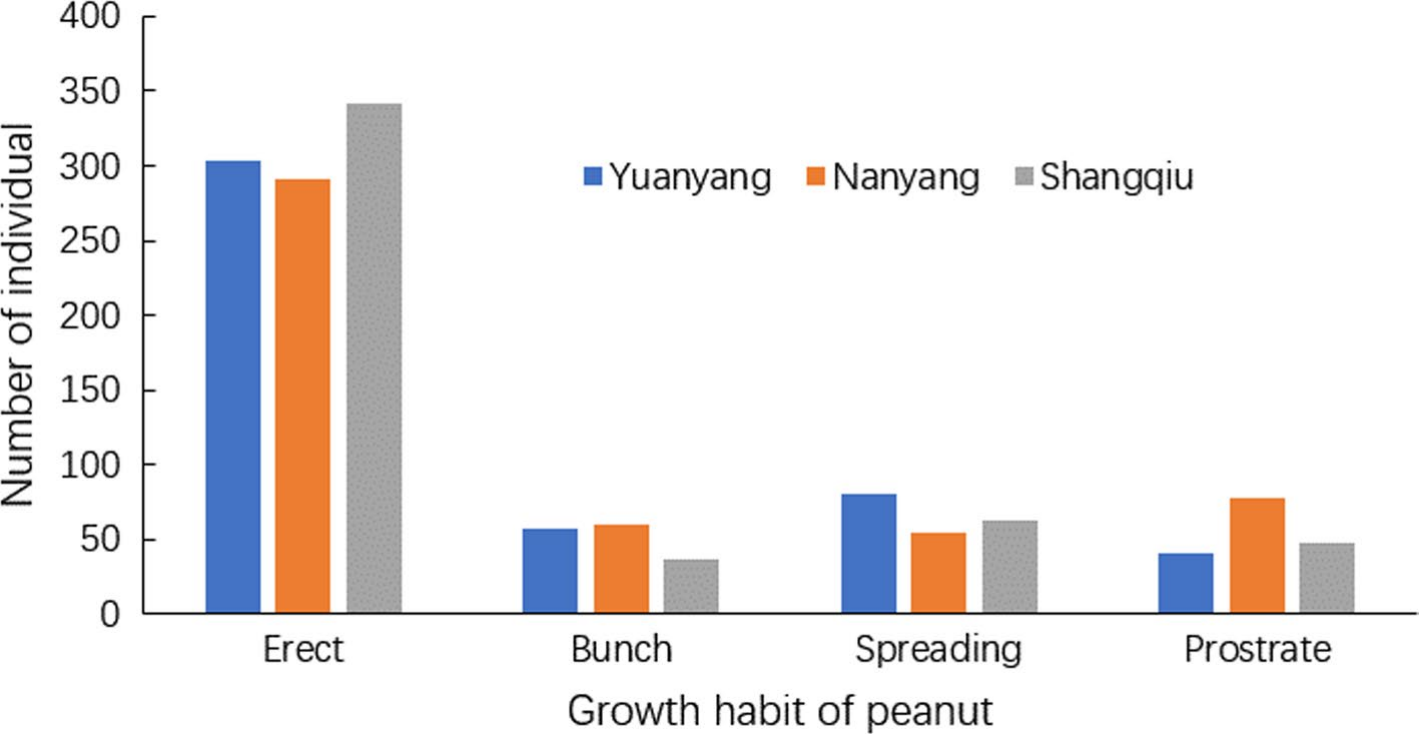
Frequency of peanut growth habits in 521 recombinant inbred lines at three growth environments including Yuanyang, Nanyang and Shangqiu

**Fig. 2:**
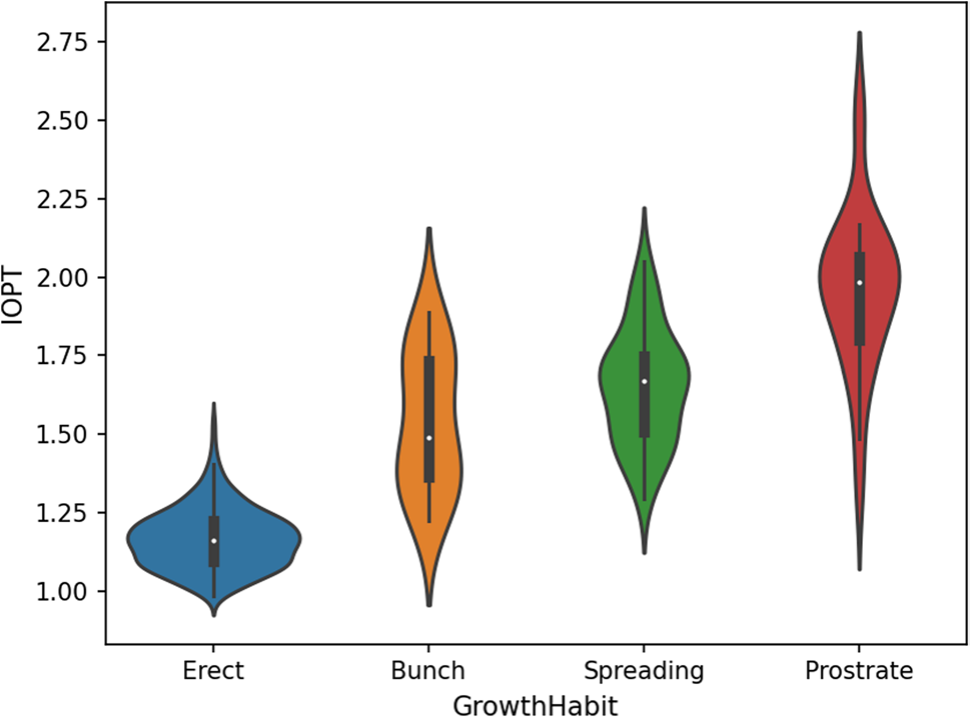
Violin plot of index of plant type (IOPT) for different growth habits in peanut RIL population

**Fig. 3:**
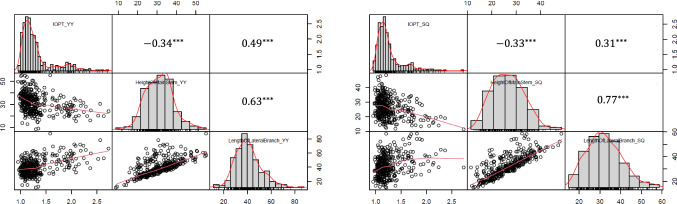
Correlation between index of plant type (IOPT), height of main stem and length of first lateral branch in Yuanza9102-derived recombinant inbred lines. **a** Phenotypic correlation at Yuanyang location (referred to as “YY” in chart); **b** phenotypic correlation at Shangqiu location (referred to as “SQ” in chart)

The ratio of the erect and bunch peanut lines to the spreading and prostrate peanuts was approximately three to one in all the three locations (Fig. 1). The observed GH traits of individual RILs may vary among the three locations, with at least one location exhibiting a distinct phenotype (Table S2 and Figure S1), indicating the complexity of the GH trait inheritance. Furthermore, the GH traits of approximately 71% of RILs were stable in three environments, and the IOPT value for each GH fell within a distinct range (Fig. 2). In general, the IOPT values of the erect peanut ranged from 1.00 to 1.25, whereas those of prostrate peanuts ranged from 1.75 to 2.25. Significantly positive correlations were found between IOPT and the length of the first lateral branch, and between the height of the main stem and the length of the first lateral branch, while a negative correlation was found between IOPT and the height of the main stem (Fig. 3).

### QTL identified for peanut growth habit traits

**Fig. 4:**
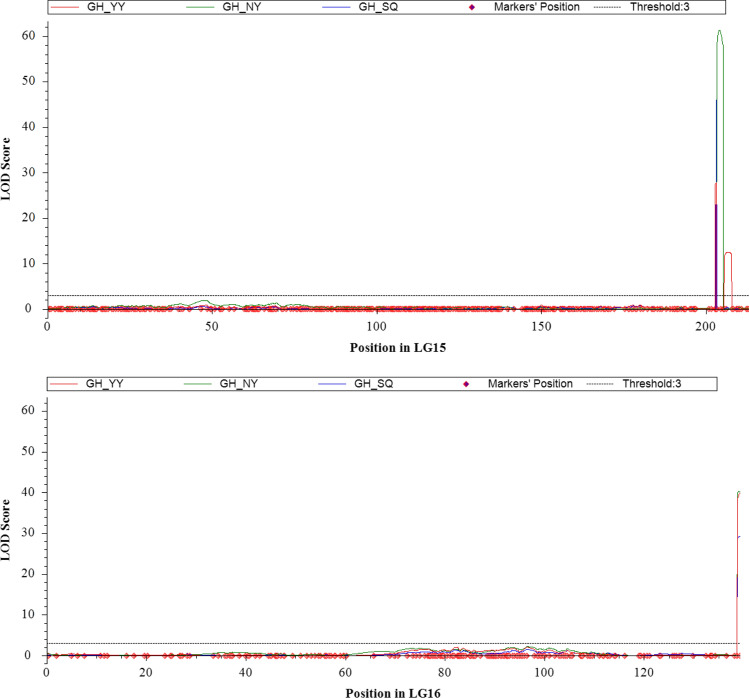
QTL mapping for peanut growth habit at three growth environments including Yuanyang (YY), Nanyang (NY) and Shangqiu (SQ)

**Fig. 5:**
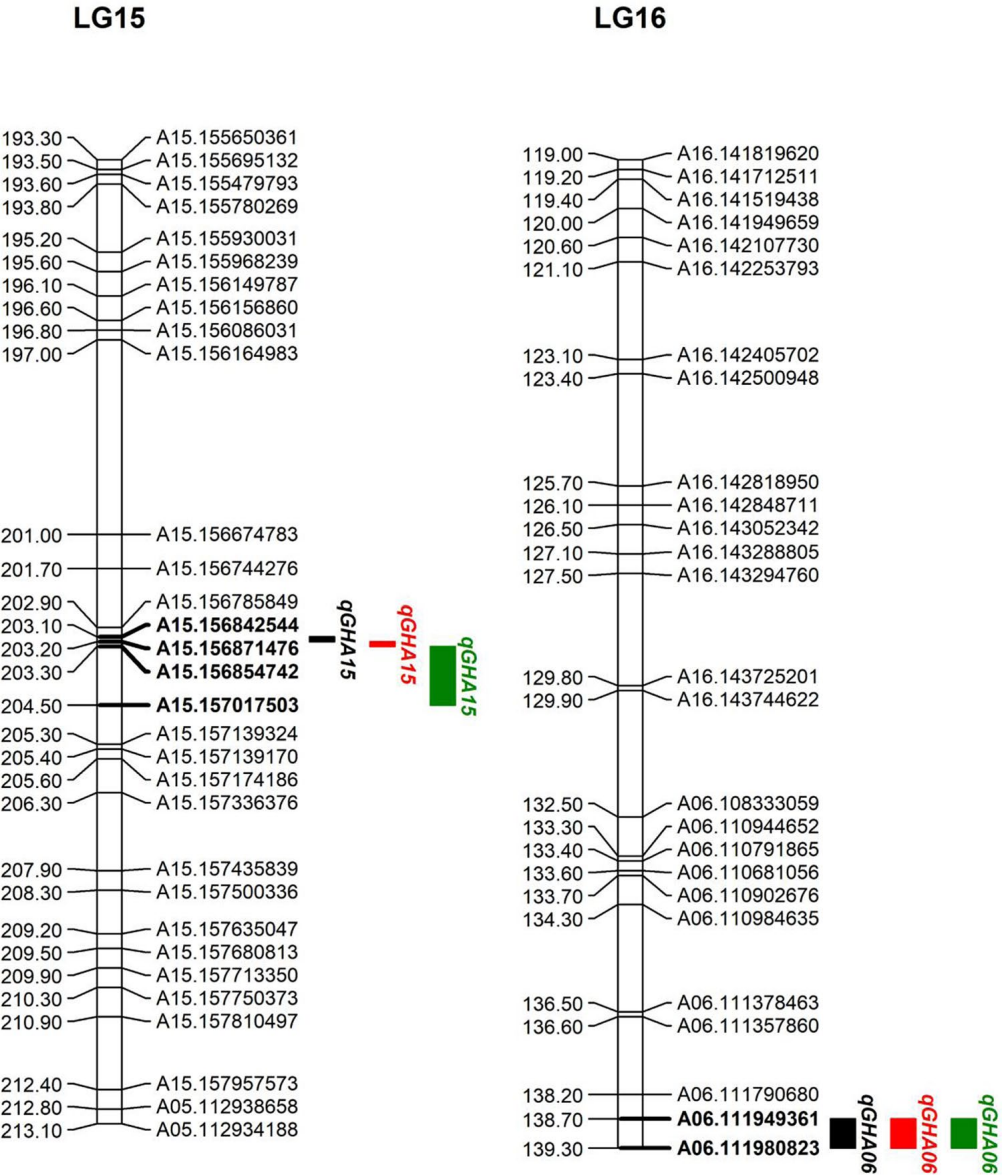
QTL for peanut growth habit identified on LG 15 and LG 16 in three different growth environments

Two major QTLs for GH with high LOD scores and PVE values on LG 15 and LG 16 were identified in three different locations (Table 1 and Fig. 4). *qGHA15* was located between 203.1 and 204.2 cM on LG 15 (Fig. 5), with the LOD score ranging from 27.76 to 61.28 and PVE over 20%. The additive effect of *qGHA15* was approximately − 0.20; hence, the SNPs from the male parent wt09-0023 could be attributable to the spreading GH trait. *qGHA06* on LG 16 was flanked by the markers A06.111949361 and A06.111980823 (Fig. 5), with LOD scores ranging between 29.24 and 40.20 and PVE ranging between 16.83 and 28.21%. The additive effect varied between 0.15 and 0.19, suggesting that SNPs from Yuanza9102 are closely associated with the spreading GH trait of peanut in the interval of *qGHA06*. In addition, QTLs for IOPT were identified in the 521 RILs in at least two locations (Table 1). As expected, *qIOPTA06* was identified on LG 16 in the same interval as *qGHA06*, whereas *qIOPTA15* was located on LG 15 next to *qGHA15.*

**Table 1:** QTL for GH and IOPT in three different growth environments

QTL	Linkage group	Position (cM)	Marker Interval	LOD	PVE (%)	Add	Environment
*qGHA15*	15	203.1–204.2	A15.156842544–A15.157017503	27.76–61.28	18.51–34.70	− 0.24 to − 0.13	YY, NY, SQ
*qGHA06*	16	139.1–139.3	A06.111949361–A06.111980823	29.24–40.20	16.83–28.21	0.15 to 0.19	YY, NY, SQ
*qIOPTA15*	15	204.5	A15.157017503–A15.157139324	38.21–39.23	23.54–23.94	− 0.17 to − 0.14	YY, SQ
*qIOPTA06*	16	138.9–139.3	A06.111949361–A06.111980823	30.45–30.69	17.82–18.34	0.12 to 0.15	YY, SQ

*LOD* logarithm of odds, *PVE* percentage of variance explained

YY, NY and SQ represent Yuanyang, Nanyang and Shangqiu, respectively

Meanwhile, QTLs with epistatic effects were investigated. It is worthy of noting that the two QTLs identified by the additive model were estimated to have significant interaction with each other (Table 2). Their epistatic effect was − 0.1, indicating that the spreading or prostrate GH in the RIL population is primarily contributed by alleles from the parent wt09-0023.

**Table 2:** Epistatic QTL for peanut growth habit traits at three different growth environments

QTL	QTL1	Marker Interval	QTL2	Marker Interval	LOD	PVE (%)	AddbyAdd	Environment
*Epi-qGH*	*qGHA15*	A15.156785849–A15.157017503	*qGHA06*	A06.111949361–A06.111980823	35.02–51.89	60.26–71.47	− 0.17 to − 0.15	YY, NY, SQ
*Epi-qIOPT*	*qIOPTA15*	A15.156854742–A15.157017503	*qIOPTA06*	A06.111949361–A06.111980823	12.04–12.52	32.34–47.10	− 0.09 to − 0.07	YY, SQ

YY, NY and SQ represent Yuanyang, Nanyang and Shangqiu, respectively

### Effects of genetic variants in the identified QTL region

By leveraging the resequencing data analysis, the effects of SNPs and INDELs in the QTL regions of LG 15 and LG 16 were predicted using the SnpEff package (Cingolani et al. 2012). Based on the annotation, four variants were polymorphic between the RIL parents and may affect genes involved in plant developmental processes (Table 3). On chromosome 15, a SNP variant at 156,854,742 bp resulted in a substitution from serine to isoleucine. It was predicted as a missense mutation in the protein coding sequence of *Arahy.QV02Z8* (annotated as gibberellin 20 oxidase 2-like). A SNP at 156,931,574 bp on chromosome 15 was located in the coding sequence of *Arahy.509QUQ* (annotated as MADS-box transcription factor 17-like), resulting in a splicing site variant. In addition, an INDEL variant (an insertion of “TA” at 156,976,352 bp) located in the coding sequence of *Arahy.ATH5WE* (annotated as MADS-box transcription factor 6) may lead to a frameshift mutation. On chromosome 6, an INDEL variant in the coding sequence of *Arahy.SC7TJM* (annotated as homeobox-leucine zipper protein) led to a conservative in-frame deletion of 3-nucleotide (ATT) repeats coding for asparagine on the reverse strand.

**Table 3:** Candidate SNP and INDEL associated with peanut growth habit

Variant	Reference allele	Alternative allele	Chromosome	Position	Gene ID	Position of genes in Tifrunner genome version 1.0		Annotation
						Chromosome	Genomic region	
SNP	C	A	15	156,854,742	Arahy.QV02Z8	Arahy.15	156,853,125 to 156,855,627 (− strand)	gibberellin 20 oxidase 2-like
SNP	A	C	15	156,931,574	Arahy.509QUQ	Arahy.15	156,929,580 to 156,936,084 (+ strand)	MADS-box transcription factor 17-like
INDEL	G	GTA	15	156,976,352	Arahy.ATH5WE	Arahy.15	156,967,808 to 156,977,402 (− strand)	MADS-box transcription factor 6
INDEL	C(ATT)_5_	C(ATT)_3_, C(ATT)_4_	6	111,973,258	Arahy.SC7TJM	Arahy.06	111,973,154 to 111,975,749 (− strand)	Homeobox-leucine zipper protein family

### Validation of molecular markers developed for growth habit in peanut

**Fig. 6:**
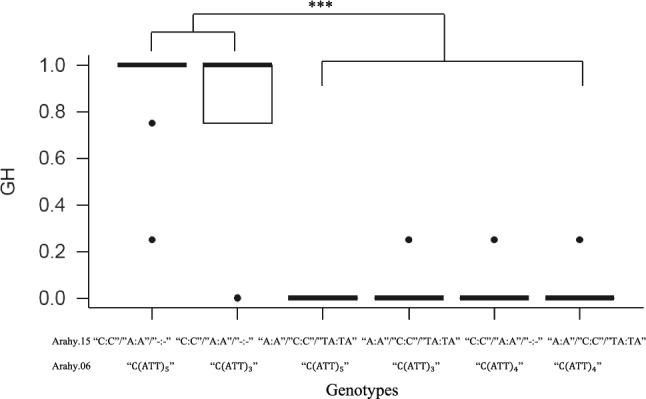
Validation of molecular markers for growth habit in 77 germplasm accessions. *Note*: Erect, bunch, spreading and prostrate growth habits were represented by 0, 0.25, 0.75 and 1, respectively. *** denotes significance at *p* value of 0.001

The three KASP markers on chromosome 15 and an INDEL marker on chromosome 6 were then examined in 165 RILs (Table S3). Among them, 55 spreading or prostrate RILs had consistent genotypes featuring chromosome 15 markers “C:C”/“A:A”/“-:-” and chromosome 6 INDEL marker C(ATT)_3_; 31 erect RILs carried the “C:C”/“A:A”/“-:-” genotype and C(ATT)_5_ INDEL genotype; and 79 erect RILs carried genotype “A:A”/“C:C”/“TA:TA” on chromosome 15 and either C(ATT)_5_ or C(ATT)_3_ as the INDEL genotype on chromosome 6. In the validation panel, 35 prostrate or spreading accessions and 42 peanut accessions with erect or bunch GH were also genotyped by these molecular markers (Fig. 6). The prostrate/spreading accessions were found to carry “C:C”/“A:A”/“-:-” on chromosome 15 and C(ATT)_5_ or C(ATT)_3_ on chromosome 6 (Table S4), whereas the erect/bunch accessions were found to feature the chromosome 15 genotype “A:A”/“C:C”/“TA:TA” and the INDEL genotype C(ATT)_5_ or C(ATT)_3_ or C(ATT)_4_. Furthermore, genotypes with “C:C”/“A:A”/“-:-” on chromosome 15 and C(ATT)_4_ on chromosome 6 were found to be closely associated with erect or bunch GH trait (Table S4).

## Discussion

Previous QTL studies in peanut for height of main stem, length of first lateral branch and number of primary branches were limited to the SSR-based low-density genetic map (Li et al. 2017; Khedikar et al. 2018). In the first study on peanut GH, a population derived from a cross between a spreading type and a bunch type peanut was investigated by bulked segregant analysis, and as a result, QTLs for peanut GH were identified on chromosome B05 in the region between 145,553,897 and 146,649,943 bp (Kayam et al. 2017). In a more recent QTL analysis, a region between 144,193,467 and 144,513,467 bp on chromosome B05 was identified to be associated with GH in peanut (Li et al. 2022). These QTLs were identified by leveraging the released sequencing database of the diploid progenitors (Bertioli et al. 2016) as the reference genome. The availability of whole genome sequence of cultivated peanut (Bertioli et al. 2019; Zhuang et al. 2019; Chen et al. 2019) has facilitated high-density marker discovery and efficient QTL mapping. In this study, QTLs for GH traits in peanut were identified in a RIL population of 521 lines and genotyped by 5120 SNP markers.

In this study, we have identified *qGHA15* as a key QTL for the GH trait, which was located on the same chromosome as those identified by previous studies (Kayam et al. 2017; Li et al. 2022). In addition, a novel stable QTL, *qGHA06*, has also been identified. Two SNPs and an INDEL for *qGHA15* and an INDEL for *qGHA06* were identified in a RIL population derived from Yuanza9102 and wt09-0023, and these markers were validated in a panel of 77 accessions. The four variants were in the protein-coding sequences of putative genes and were predicted to have moderate or high impact on the candidate genes. A SNP was identified in the coding sequence of *Arahy.QV02Z8* that encodes a gibberellin 20 oxidase 2-like protein. In rice, the semi-dwarf gene (*sd1*) was found to correspond to a defective gibberellin 20 oxidase 2 (GA20ox2) (Spielmeyer et al. 2002). A SNP and an INDEL were identified in *Arahy.509QUQ* and *Arahy.ATH5WE* on chromosome 15, which are both MADS-box transcription factors and may be involved in regulating gene expression throughout plant development. An INDEL for *qGHA06* was identified in the coding sequence of *Arahy.SC7TJM* on chromosome 6. *Arahy.SC7TJM* encodes a homeobox-leucine zipper protein that was reported to regulate tiller angle in rice (Hu et al. 2020). *Arahy.SC7TJM* was positioned on chromosome 16 based on Tifrunner version 2.0 gene models (www.peanutbase.org), in accordance with LG 16 of the genetic linkage map where the INDEL marker for *qGHA06* was assigned to.

In this study, four markers closely linked with peanut GH were developed and validated in both the RIL population and a validation panel. The genotypes of three KASP markers on chromosome 15 were strongly correlated with one another, either with “C:C”/“A:A”/“-:-” or “A:A”/“C:C”/“TA:TA”. Notably, peanut accessions with spreading or prostrate GH were exclusively “C:C”/“A:A”/“-:-” for the three markers on chromosome 15. Peanut accessions carrying the “A:A”/“C:C”/“TA:TA” markers on chromosome 15 combined with C(ATT)_5_ or C(ATT)_3_ INDEL marker on chromosome 6 were identified in the erect and bunch GH groups. In addition, the genotypes with “C:C”/“A:A”/“-:-” on chromosome 15 combined with C(ATT)_3_ on chromosome 6 were closely associated with the spreading and prostrate GH. The INDEL genotype on chromosome 6, C(ATT)_4_, was unique to the erect and bunch peanut accessions in the validation panel consisting of 77 germplasm accessions (Table S4). In the 165 RILs that were selected for genotyping, the C(ATT)_4_ allele was not identified because of its absence in their parents. Instead, the genotypes of 31 erect peanuts among the 165 RILs were “C:C”/“A:A”/“-:-” on chromosome 15 and C(ATT)_5_ on chromosome 6, consistent with the parent wt09-0023. However, in the validation panel, “C:C”/“A:A”/“-:-” on chromosome 15 combined with C(ATT)_5_ on chromosome 6 were solely identified in the spreading and prostrate accessions. Overall, the two QTLs identified in this study have proven to be useful in fine mapping for the GH traits, offering promise for further identification of novel QTLs for GH traits in populations with different genetic backgrounds.

Further, in a number of prior QTL studies, IOPT was used as an indicator of peanut GH (Li et al. 2019, 2022). The violin plot of the four types of GH traits in the RIL population used in this study revealed a correlation between IOPT and peanut GH (Fig. 2). *qIOPTA15* was found to be adjacent to *qGHA15* on LG 15, while *qIOPTA06* was colocalized with *qGHA06* on LG 16 (Table 1). These two major QTLs were demonstrated to be stable across three different environments.

In conclusion, in this study we have developed and validated four molecular markers for peanut GH traits. These markers could not only be directly applied to distinguish the peanut types with spreading and prostrate GH traits from those with erect and bunch traits in peanut breeding, but also pave the way for further analysis of other candidate genes underpinning GH traits in peanut.

## Supplementary Information

Below is the link to the electronic supplementary material.

Supplementary Figure 1 Distribution of residuals for peanut growth habit in the RIL population at three different growth environments, including Yuanyang (YY), Nanyang (NY) and Shangqiu (SQ).

Supplementary tables

## Data Availability

The data generated and analyzed in this study are included in the manuscript and supplementary files.
