# Supplementary figures and images for: Identification of quantitative trait loci and development of diagnostic markers for growth habit traits in peanut (Arachis hypogaea L.)
Source: Theor Appl Genet. 2023 Apr 7;136(5):105. doi: 10.1007/s00122-023-04327-9 (PMC10082100; doi:10.1007/s00122-023-04327-9)

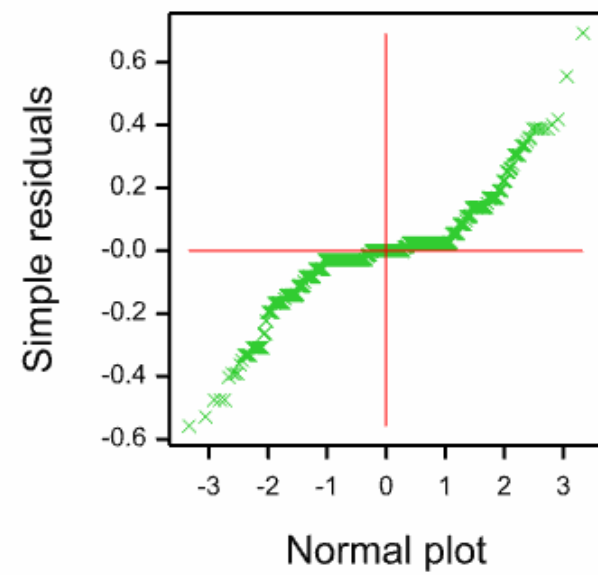

Supplement: Supplementary file 1 — Supplementary Figure 1 Distribution of residuals for peanut growth habit in the RIL population at three different growth environments, including Yuanyang (YY), Nanyang (NY) and Shangqiu (SQ). [file 122_2023_4327_MOESM1_ESM.pdf]
