# Supplementary material for: Identification of quantitative trait loci and development of diagnostic markers for growth habit traits in peanut (Arachis hypogaea L.)
Source: Theor Appl Genet. 2023 Apr 7;136(5):105. doi: 10.1007/s00122-023-04327-9 (PMC10082100; doi:10.1007/s00122-023-04327-9)
Supplement: Supplementary file 2 — Supplementary tables [file 122_2023_4327_MOESM2_ESM.docx]

**Supplementary Table S1 Primer sequences of four diagnostic markers developed for peanut growth habit trait**

| **Chromosome** | **Position** | **Variant** | **Type** | **Primer Seq Allele X** | **Primer Seq Allele Y** | **Primer Seq common** |
| --- | --- | --- | --- | --- | --- | --- |
| Arahy.15 | 156854742 | C/A | SNP | GTATTCCTTGACAATCTTTGGTGAGC | GTATTCCTTGACAATCTTTGGTGAGA | CTTCCATGGAAGGAGACTCTCTCTT |
| Arahy.15 | 156931574 | A/C | SNP | ATTATTATTATTGGTGATGTGCCTTGCA | ATTATTATTATTGGTGATGTGCCTTGCC | GTTCGCTCCAGAGATTCATATTTTACCTT |
| Arahy.15 | 156976352 | -/TA | INDEL | GAGGCTCTTTGAATACTCAAGTAC | GAGGCTCTTTGAATACTCAAGTAT | TAAAGTGATTAAGTAAGGGCTTACTCTGAA |
| Arahy.06 | 111973258 | C(ATT)_5_/C(ATT)_3_,C(ATT)_4_ | INDEL | Forward primer: TGTTGCGGATGATGAGTGTT | Reverse primer: TGCCCTTCTTGTGAGACAAT |  |

**Supplementary Table S2 ANOVA for growth habit trait in year 2021**

| **Source** | **DF** | **SS** | **MS** | **F-value** | **Pr>F** | **EstimatedVar** |
| --- | --- | --- | --- | --- | --- | --- |
| Model | 520 | 188.514 | 0.363 | 25.265 | 0.00E+00 |  |
| Error | 935 | 13.416 | 0.014 |  |  |  |
| Total | 1455 | 201.930 |  |  |  |  |
| R-square | 0.934 |  |  |  |  |  |
| Location | 2 | 0.974 | 0.487 | 33.940 | 1.90E-05 | 0.001 |
| RIL | 518 | 187.540 | 0.362 | 25.232 | 0.00E+00 | 0.124 |

**Supplementary Table S3 Genotypes of 165 RILs revealed by four markers developed for growth habit trait in peanut**

| **RIL** | **Arahy15.156854742** | **Arahy15.156931574** | **Arahy15.156976352** | **Arahy06.111973258** | **GH** |
| --- | --- | --- | --- | --- | --- |
| P271 | A:A | C:C | TA:TA | C(ATT)_5_ | Erect |
| P273 | A:A | C:C | TA:TA | C(ATT)_5_ | Erect |
| P280 | A:A | C:C | TA:TA | C(ATT)_5_ | Erect |
| P287 | A:A | C:C | TA:TA | C(ATT)_5_ | Erect |
| P306 | A:A | C:C | TA:TA | C(ATT)_5_ | Erect |
| P324 | A:A | C:C | TA:TA | C(ATT)_5_ | Erect |
| P331 | A:A | C:C | TA:TA | C(ATT)_5_ | Erect |
| P343 | A:A | C:C | TA:TA | C(ATT)_5_ | Erect |
| P369 | A:A | C:C | TA:TA | C(ATT)_5_ | Erect |
| P374 | A:A | C:C | TA:TA | C(ATT)_5_ | Erect |
| P394 | A:A | C:C | TA:TA | C(ATT)_5_ | Erect |
| P404 | A:A | C:C | TA:TA | C(ATT)_5_ | Erect |
| P439 | A:A | C:C | TA:TA | C(ATT)_5_ | Erect |
| P446 | A:A | C:C | TA:TA | C(ATT)_5_ | Erect |
| P456 | A:A | C:C | TA:TA | C(ATT)_5_ | Erect |
| P457 | A:A | C:C | TA:TA | C(ATT)_5_ | Erect |
| P458 | A:A | C:C | TA:TA | C(ATT)_5_ | Erect |
| P470 | A:A | C:C | TA:TA | C(ATT)_5_ | Erect |
| P474 | A:A | C:C | TA:TA | C(ATT)_5_ | Erect |
| P475 | A:A | C:C | TA:TA | C(ATT)_5_ | Erect |
| P477 | A:A | C:C | TA:TA | C(ATT)_5_ | Erect |
| P480 | A:A | C:C | TA:TA | C(ATT)_5_ | Erect |
| P490 | A:A | C:C | TA:TA | C(ATT)_5_ | Erect |
| P514 | A:A | C:C | TA:TA | C(ATT)_5_ | Erect |
| P516 | A:A | C:C | TA:TA | C(ATT)_5_ | Erect |
| P518 | A:A | C:C | TA:TA | C(ATT)_5_ | Erect |
| P520 | A:A | C:C | TA:TA | C(ATT)_5_ | Erect |
| P522 | A:A | C:C | TA:TA | C(ATT)_5_ | Erect |
| P536 | A:A | C:C | TA:TA | C(ATT)_5_ | Erect |
| P540 | A:A | C:C | TA:TA | C(ATT)_5_ | Erect |
| P542 | A:A | C:C | TA:TA | C(ATT)_5_ | Erect |
| P549 | A:A | C:C | TA:TA | C(ATT)_5_ | Erect |
| P553 | A:A | C:C | TA:TA | C(ATT)_5_ | Erect |
| P556 | A:A | C:C | TA:TA | C(ATT)_5_ | Erect |
| P557 | A:A | C:C | TA:TA | C(ATT)_5_ | Erect |
| P559 | A:A | C:C | TA:TA | C(ATT)_5_ | Erect |
| P563 | A:A | C:C | TA:TA | C(ATT)_5_ | Erect |
| P567 | A:A | C:C | TA:TA | C(ATT)_5_ | Erect |
| P568 | A:A | C:C | TA:TA | C(ATT)_5_ | Erect |
| P581 | A:A | C:C | TA:TA | C(ATT)_5_ | Erect |
| P582 | A:A | C:C | TA:TA | C(ATT)_5_ | Erect |
| P586 | A:A | C:C | TA:TA | C(ATT)_5_ | Erect |
| P592 | A:A | C:C | TA:TA | C(ATT)_5_ | Erect |
| P593 | A:A | C:C | TA:TA | C(ATT)_5_ | Erect |
| P594 | A:A | C:C | TA:TA | C(ATT)_5_ | Erect |
| P610 | A:A | C:C | TA:TA | C(ATT)_5_ | Erect |
| P266 | C:C | A:A | -:- | C(ATT)_5_ | Erect |
| P270 | C:C | A:A | -:- | C(ATT)_5_ | Erect |
| P294 | C:C | A:A | -:- | C(ATT)_5_ | Erect |
| P296 | C:C | A:A | -:- | C(ATT)_5_ | Erect |
| P297 | C:C | A:A | -:- | C(ATT)_5_ | Erect |
| P304 | C:C | A:A | -:- | C(ATT)_5_ | Erect |
| P318 | C:C | A:A | -:- | C(ATT)_5_ | Erect |
| P322 | C:C | A:A | -:- | C(ATT)_5_ | Erect |
| P332 | C:C | A:A | -:- | C(ATT)_5_ | Erect |
| P338 | C:C | A:A | -:- | C(ATT)_5_ | Erect |
| P347 | C:C | A:A | -:- | C(ATT)_5_ | Erect |
| P393 | C:C | A:A | -:- | C(ATT)_5_ | Erect |
| P397 | C:C | A:A | -:- | C(ATT)_5_ | Erect |
| P403 | C:C | A:A | -:- | C(ATT)_5_ | Erect |
| P419 | C:C | A:A | -:- | C(ATT)_5_ | Erect |
| P422 | C:C | A:A | -:- | C(ATT)_5_ | Erect |
| P427 | C:C | A:A | -:- | C(ATT)_5_ | Erect |
| P440 | C:C | A:A | -:- | C(ATT)_5_ | Erect |
| P444 | C:C | A:A | -:- | C(ATT)_5_ | Erect |
| P466 | C:C | A:A | -:- | C(ATT)_5_ | Erect |
| P468 | C:C | ? | -:- | C(ATT)_5_ | Erect |
| P473 | C:C | A:A | -:- | C(ATT)_5_ | Erect |
| P504 | C:C | A:A | -:- | C(ATT)_5_ | Erect |
| P596 | C:C | A:A | -:- | C(ATT)_5_ | Erect |
| P604 | C:C | A:A | -:- | C(ATT)_5_ | Erect |
| P611 | C:C | A:A | -:- | C(ATT)_5_ | Erect |
| P613 | C:C | A:A | -:- | C(ATT)_5_ | Erect |
| P625 | C:C | A:A | -:- | C(ATT)_5_ | Erect |
| P628 | C:C | A:A | -:- | C(ATT)_5_ | Erect |
| P630 | C:C | A:A | -:- | C(ATT)_5_ | Erect |
| P633 | C:C | A:A | -:- | C(ATT)_5_ | Erect |
| P290 | A:A | C:C | TA:TA | C(ATT)_3_ | Erect |
| P299 | A:A | C:C | TA:TA | C(ATT)_3_ | Erect |
| P312 | A:A | C:C | TA:TA | C(ATT)_3_ | Erect |
| P325 | A:A | C:C | TA:TA | C(ATT)_3_ | Erect |
| P327 | A:A | C:C | TA:TA | C(ATT)_3_ | Erect |
| P330 | A:A | C:C | TA:TA | C(ATT)_3_ | Erect |
| P345 | A:A | C:C | TA:TA | C(ATT)_3_ | Erect |
| P348 | A:A | C:C | TA:TA | C(ATT)_3_ | Erect |
| P360 | A:A | C:C | TA:TA | C(ATT)_3_ | Erect |
| P361 | A:A | C:C | TA:TA | C(ATT)_3_ | Erect |
| P373 | A:A | C:C | TA:TA | C(ATT)_3_ | Erect |
| P375 | A:A | C:C | TA:TA | C(ATT)_3_ | Erect |
| P380 | A:A | C:C | TA:TA | C(ATT)_3_ | Erect |
| P387 | A:A | C:C | TA:TA | C(ATT)_3_ | Erect |
| P395 | A:A | C:C | TA:TA | C(ATT)_3_ | Erect |
| P400 | A:A | C:C | TA:TA | C(ATT)_3_ | Erect |
| P401 | A:A | C:C | TA:TA | C(ATT)_3_ | Erect |
| P425 | A:A | C:C | TA:TA | C(ATT)_3_ | Erect |
| P442 | A:A | C:C | TA:TA | C(ATT)_3_ | Erect |
| P478 | A:A | C:C | TA:TA | C(ATT)_3_ | Erect |
| P484 | A:A | C:C | TA:TA | C(ATT)_3_ | Erect |
| P487 | A:A | C:C | TA:TA | C(ATT)_3_ | Erect |
| P494 | A:A | C:C | TA:TA | C(ATT)_3_ | Erect |
| P499 | A:A | C:C | TA:TA | C(ATT)_3_ | Erect |
| P506 | A:A | C:C | TA:TA | C(ATT)_3_ | Erect |
| P512 | A:A | C:C | TA:TA | C(ATT)_3_ | Erect |
| P517 | A:A | C:C | TA:TA | C(ATT)_3_ | Erect |
| P548 | A:A | C:C | TA:TA | C(ATT)_3_ | Erect |
| P580 | A:A | C:C | TA:TA | C(ATT)_3_ | Erect |
| P600 | A:A | C:C | TA:TA | C(ATT)_3_ | Erect |
| P602 | A:A | C:C | TA:TA | C(ATT)_3_ | Erect |
| P634 | A:A | C:C | TA:TA | C(ATT)_3_ | Erect |
| P316 | C:C | C:C | TA:TA | C(ATT)_3_ | Erect |
| P278 | C:C | A:A | -:- | C(ATT)_3_ | Spreading |
| P283 | C:C | A:A | -:- | C(ATT)_3_ | Spreading |
| P329 | C:C | A:A | -:- | C(ATT)_3_ | Spreading |
| P336 | C:C | A:A | -:- | C(ATT)_3_ | Spreading |
| P355 | C:C | A:A | -:- | C(ATT)_3_ | Spreading |
| P357 | C:C | A:A | -:- | C(ATT)_3_ | Spreading |
| P372 | C:C | A:A | -:- | C(ATT)_3_ | Spreading |
| P381 | C:C | A:A | -:- | C(ATT)_3_ | Spreading |
| P396 | C:C | A:A | -:- | C(ATT)_3_ | Spreading |
| P418 | C:C | A:A | -:- | C(ATT)_3_ | Spreading |
| P437 | C:C | A:A | -:- | C(ATT)_3_ | Spreading |
| P438 | C:C | A:A | -:- | C(ATT)_3_ | Spreading |
| P488 | C:C | A:A | -:- | C(ATT)_3_ | Spreading |
| P498 | C:C | A:A | -:- | C(ATT)_3_ | Spreading |
| P526 | C:C | A:A | -:- | C(ATT)_3_ | Spreading |
| P574 | C:C | A:A | -:- | C(ATT)_3_ | Spreading |
| P644 | C:C | ? | -:- | C(ATT)_3_ | Spreading |
| P650 | C:C | A:A | -:- | C(ATT)_3_ | Spreading |
| P672 | C:C | A:A | -:- | C(ATT)_3_ | Spreading |
| P681 | C:C | A:A | -:- | C(ATT)_3_ | Spreading |
| P682 | C:C | A:A | -:- | C(ATT)_3_ | Spreading |
| P728 | C:C | A:A | -:- | C(ATT)_3_ | Spreading |
| P739 | C:C | A:A | -:- | C(ATT)_3_ | Spreading |
| P755 | C:C | A:A | -:- | C(ATT)_3_ | Spreading |
| P760 | C:C | A:A | -:- | C(ATT)_3_ | Spreading |
| P765 | C:C | A:A | -:- | C(ATT)_3_ | Spreading |
| P787 | C:C | A:A | -:- | C(ATT)_3_ | Spreading |
| P740 | A:A | C:C | -:- | C(ATT)_3_ | Spreading |
| P366 | A:A | A:A | -:- | C(ATT)_3_ | Prostrate |
| P262 | C:C | A:A | -:- | C(ATT)_3_ | Prostrate |
| P288 | C:C | A:A | -:- | C(ATT)_3_ | Prostrate |
| P305 | C:C | A:A | -:- | C(ATT)_3_ | Prostrate |
| P308 | C:C | A:A | -:- | C(ATT)_3_ | Prostrate |
| P309 | C:C | A:A | -:- | C(ATT)_3_ | Prostrate |
| P315 | C:C | A:A | -:- | C(ATT)_3_ | Prostrate |
| P323 | C:C | A:A | -:- | C(ATT)_3_ | Prostrate |
| P335 | C:C | A:A | -:- | C(ATT)_3_ | Prostrate |
| P365 | C:C | A:A | -:- | C(ATT)_3_ | Prostrate |
| P368 | C:C | A:A | -:- | C(ATT)_3_ | Prostrate |
| P409 | C:C | A:A | -:- | C(ATT)_3_ | Prostrate |
| P424 | C:C | A:A | -:- | C(ATT)_3_ | Prostrate |
| P433 | C:C | A:A | -:- | C(ATT)_3_ | Prostrate |
| P460 | C:C | A:A | -:- | C(ATT)_3_ | Prostrate |
| P489 | C:C | A:A | -:- | C(ATT)_3_ | Prostrate |
| P530 | C:C | A:A | -:- | C(ATT)_3_ | Prostrate |
| P538 | C:C | A:A | -:- | C(ATT)_3_ | Prostrate |
| P539 | C:C | ? | -:- | C(ATT)_3_ | Prostrate |
| P616 | C:C | A:A | -:- | C(ATT)_3_ | Prostrate |
| P620 | C:C | A:A | -:- | C(ATT)_3_ | Prostrate |
| P648 | C:C | A:A | -:- | C(ATT)_3_ | Prostrate |
| P657 | C:C | A:A | -:- | C(ATT)_3_ | Prostrate |
| P742 | C:C | A:A | -:- | C(ATT)_3_ | Prostrate |
| P747 | C:C | A:A | -:- | C(ATT)_3_ | Prostrate |
| P749 | C:C | A:A | -:- | C(ATT)_3_ | Prostrate |
| P795 | C:C | A:A | -:- | C(ATT)_3_ | Prostrate |

**Supplementary Table S4 Validation panel genotyped by markers developed for growth habit trait in peanut**

| **Accession** | **Origin** | **Classification** | **Arahy15.156854742** | **Arahy15.156931574** | **Arahy15.156976352** | **Arahy06.111973258** | **GH** |
| --- | --- | --- | --- | --- | --- | --- | --- |
| N612 | China | landrace | NA | NA | -:- | C(ATT)_3_ | Prostrate |
| N629 | Mexico | NA | C:C | A:A | -:- | C(ATT)_3_ | Prostrate |
| N663 | China | landrace | NA | NA | -:- | C(ATT)_3_ | Prostrate |
| N665 | China | landrace | C:C | A:A | -:- | C(ATT)_3_ | Prostrate |
| N666 | Mexico | NA | C:C | NA | -:- | C(ATT)_3_ | Prostrate |
| N675 | China | landrace | NA | NA | -:- | C(ATT)_3_ | Prostrate |
| N679 | China | landrace | C:C | A:A | -:- | C(ATT)_3_ | Prostrate |
| N687 | U.S | released variety | C:C | A:A | -:- | C(ATT)_3_ | Prostrate |
| N525 | China | landrace | NA | NA | NA | C(ATT)_3_ | Prostrate |
| N614 | China | landrace | NA | NA | NA | C(ATT)_3_ | Prostrate |
| N633 | China | landrace | NA | NA | -:- | C(ATT)_3_ | Spreading |
| N635 | China | landrace | C:C | A:A | -:- | C(ATT)_3_ | Spreading |
| N662 | China | landrace | C:C | A:A | -:- | C(ATT)_3_ | Spreading |
| N683 | China | landrace | C:C | A:A | -:- | C(ATT)_3_ | Spreading |
| N739 | U.S | released variety | C:C | A:A | -:- | C(ATT)_3_ | Spreading |
| N815 | U.S | released variety | NA | NA | -:- | C(ATT)_3_ | Spreading |
| N521 | China | landrace | NA | NA | NA | C(ATT)_3_ | Spreading |
| N558 | China | landrace | NA | NA | NA | C(ATT)_3_ | Spreading |
| N603 | China | landrace | NA | NA | NA | C(ATT)_3_ | Spreading |
| N641 | Pakistan | NA | C:C | A:A | -:- | C(ATT)_3_ | Prostrate |
| N496 | Argentina | NA | NA | NA | -:- | C(ATT)_5_ | Spreading |
| N487 | China | landrace | C:C | A:A | -:- | C(ATT)_5_ | Prostrate |
| N563 | China | NA | C:C | NA | -:- | C(ATT)_5_ | Prostrate |
| N582 | NA | NA | C:C | A:A | -:- | C(ATT)_5_ | Prostrate |
| N594 | China | landrace | C:C | A:A | -:- | C(ATT)_5_ | Prostrate |
| N628 | India | NA | C:C | A:A | -:- | C(ATT)_5_ | Prostrate |
| N649 | Madagascar | NA | C:C | A:A | -:- | C(ATT)_5_ | Prostrate |
| N659 | India | NA | C:C | A:A | -:- | C(ATT)_5_ | Prostrate |
| N670 | Cuba | NA | C:C | A:A | -:- | C(ATT)_5_ | Prostrate |
| N731 | U.S | released variety | C:C | A:A | -:- | C(ATT)_5_ | Prostrate |
| N738 | U.S | released variety | NA | A:A | -:- | C(ATT)_5_ | Prostrate |
| N816 | U.S | released variety | NA | NA | NA | C(ATT)_5_ | Prostrate |
| N532 | China | landrace | NA | A:A | -:- | C(ATT)_9_ | Spreading |
| N639 | Senegal | NA | C:C | NA | -:- | C | Prostrate |
| N462 | Argentina | NA | A:A | C:C | TA:TA | C(ATT)_5_ | Erect |
| N776 | China | released variety | A:A | C:C | TA:TA | C(ATT)_5_ | Erect |
| N713 | China | released variety | A:A | C:C | TA:TA | C(ATT)_3_ | Bunch |
| N694 | China | released variety | A:A | C:C | TA:TA | C(ATT)_3_ | Erect |
| N730 (Yuanza9102) | China | released variety | A:A | C:C | TA:TA | C(ATT)_3_ | Erect |
| N498 | China | released variety | A:A | C:C | TA:TA | C(ATT)_3_ | Erect |
| N586 | China | released variety | A:A | C:C | TA:TA | C(ATT)_3_ | Erect |
| N621 | China | released variety | A:A | C:C | TA:TA | C(ATT)_3_ | Erect |
| N489 | China | released variety | NA | NA | TA:TA | C(ATT)_3_ | Erect |
| N473 | China | released variety | A:A | C:C | TA:TA | C(ATT)_3_ | Erect |
| N689 | China | released variety | A:A | C:C | TA:TA | C(ATT)_3_ | Erect |
| N705 | China | released variety | NA | NA | TA:TA | C(ATT)_3_ | Erect |
| N704 | China | released variety | A:A | C:C | TA:TA | C(ATT)_3_ | Erect |
| N714 | China | released variety | A:A | C:C | TA:TA | C(ATT)_3_ | Erect |
| N695 | China | released variety | A:A | C:C | TA:TA | C(ATT)_3_ | Erect |
| N590 | China | released variety | A:A | C:C | TA:TA | C(ATT)_3_ | Erect |
| N842 | China | released variety | A:A | C:C | TA:TA | C(ATT)_3_ | Erect |
| N830 | China | released variety | NA | NA | TA:TA | C(ATT)_3_ | Erect |
| N607 | China | released variety | A:A | C:C | TA:TA | C(ATT)_3_ | Erect |
| N574 | China | released variety | A:A | C:C | TA:TA | C(ATT)_3_ | Erect |
| N481 | China | released variety | A:A | C:C | TA:TA | C(ATT)_3_ | Erect |
| N698 | China | released variety | A:A | C:C | TA:TA | C(ATT)_3_ | Erect |
| N615 | Argentina | NA | A:A | NA | NA | C(ATT)_4_ | Erect |
| N434 | Argentina | NA | NA | NA | NA | C(ATT)_4_ | Erect |
| N455 | Argentina | NA | NA | NA | NA | C(ATT)_4_ | Erect |
| N847 | China | released variety | A:A | C:C | TA:TA | C(ATT)_4_ | Bunch |
| N452 | Argentina | NA | A:A | C:C | TA:TA | C(ATT)_4_ | Erect |
| N564 | Argentina | NA | A:A | C:C | TA:TA | C(ATT)_4_ | Erect |
| N548 | Argentina | NA | A:A | C:C | TA:TA | C(ATT)_4_ | Erect |
| N618 | Bolivia | NA | C:C | A:A | -:- | C(ATT)_4_ | Bunch |
| N691 | China | released variety | C:C | A:A | -:- | C(ATT)_4_ | Erect |
| N518 | China | released variety | C:C | A:A | -:- | C(ATT)_4_ | Erect |
| N851 | China | released variety | C:C | A:A | -:- | C(ATT)_4_ | Erect |
| N734 | China | released variety | C:C | A:A | -:- | C(ATT)_4_ | Erect |
| N501 | China | released variety | C:C | A:A | -:- | C(ATT)_4_ | Erect |
| N438 | Brazil | NA | C:C | A:A | -:- | C(ATT)_4_ | Erect |
| N549 | Argentina | NA | C:C | A:A | -:- | C(ATT)_4_ | Erect |
| N454 | Argentina | NA | C:C | NA | -:- | C(ATT)_4_ | Erect |
| N543 | Bolivia | NA | C:C | A:A | -:- | C(ATT)_4_ | Erect |
| N790 | China | released variety | NA | NA | -:- | C(ATT)_3_ | Erect |
| N470 | China | released variety | C:C | A:A | -:- | C(ATT)_3_ | Erect |
| N750 | China | released variety | NA | NA | NA | C(ATT)_3_ | Erect |
| N735 (wt09-0023) | U.S | released variety | NA | NA | -:- | C(ATT)_5_ | Bunch |
